# Na[GeF_5_]·2HF: the first quarternary phase in the H–Na–Ge–F system

**DOI:** 10.1107/S2053229624006338

**Published:** 2024-07-10

**Authors:** Valentin Bockmair, Constantin Hoch, Irina Schusterbauer, Andreas J. Kornath

**Affiliations:** a Department Chemie, Ludwig-Maximilians Universität München, Butenandtstrasse 5-13 (Haus D), D-81377 München, Germany; University of North Texas at Dallas, USA

**Keywords:** *trans*-penta­fluoro­germanate, penta­gonal bipyramidal coordination, sodium fluoro­germanate, superacid, levelling effect, fluorine chemistry, crystal structure

## Abstract

The structure of *cis*- or *trans*-bridged [GeF_5_]^−^ anionic chains have been investigated [Mallouk *et al.* (1984). *Inorg. Chem.***23**, 3160–3166] showing the first crystal structures of μ-F-bridged penta­fluoro­germanates. Herein, we report the second crystal structure of *trans*-penta­fluoro­germanate anions present in the crystal structure of sodium *trans*-penta­fluoro­germanate(IV) bis­(hy­dro­gen fluoride), Na[GeF_5_]·2HF. The crystal structure [ortho­rhom­bic *Pca*2_1_, *a* = 12.3786 (3), *b* = 7.2189 (2), *c* = 11.4969 (3) Å and *Z* = 8] is built up from infinite chains of *trans*-linked [GeF_6_]^2−^ octa­hedra, extending along the *b* axis and spanning a network of penta­gonal bipyramidal distorted Na-centred polyhedra. These [NaF_7_] polyhedra are linked in a *trans*-edge fashion *via* hy­dro­gen fluoride mol­ecules, in analogy to already known sodium hy­dro­gen fluorides and potassium hy­dro­gen fluorides.

## Abstract 2

*trans*-Penta­fluoro­germanate di­hy­dro­gen fluoride represents the first quarternary phase in the system H–Na–Ge–F, and the third Na–Ge–F phase after the X-ray structure analyses of Na_2_GeF_6_ and CsNaGeF_6_. The crystal structure of *trans*-penta­fluoro­germanate di­hy­dro­gen fluoride consists of chains of [GeF_6_] octa­hedra and chains of penta­gonal bipyramidal [NaF_7_] polyhedra. The Raman and IR spectra show the expected vibrational modes.

## Introduction

Superacid chemistry can be applied as a powerful tool to isolate reactive volatile species by the formation of salts (Bayer *et al.*, 2022 ▸; Leitz *et al.*, 2018 ▸, 2019 ▸). These salts are mainly stabilized by F-atom inter­actions and are therefore more stable com­pared with the starting material. Furthermore, this offers the opportunity to estimate the acidity of com­pounds and structural parameters while widely retaining mol­ecular corpus (Seelbinder *et al.*, 2010 ▸).

Experiments and quantum chemical calculations revealed that the protonation of thio­sulfuric acid is successful in the superacidic system HF/*M*F_5_ (*M* = As, Sb) (Hopfinger *et al.*, 2018 ▸). Investigations of the less acidic binary superacidic sys­tem HF/GeF_4_ were performed to explore the structural chemistry of thio­sulfuric acid and its protonated species. Since the *H*_0_ value of the binary superacidic system HF/GeF_4_ was assumed to be only slightly greater than for HF/AsF_5_-based systems, monoprotonation was expected.

It turned out that the reaction of sodium thio­sulfate in HF/GeF_4_ led to the formation of Na[GeF_5_]·2HF instead of protonation of thio­sulfuric acid (see Scheme 1). Whereas no conversion of the sodium salts with the weakly coordinating anions [AsF_6_]^−^ and [SbF_6_]^−^ has been observed, the Lewis acid GeF_4_ reacts with the formation of its sodium salt, *i.e.* Na[GeF_5_].

The obtained com­pound Na[GeF_5_]·2HF is the first qua­ter­nary phase in the Na–Ge–H–F system. The crystal structure shows an unusual penta­gonal bipyramidal coordination of Na by F, in analogy to IF_7_ (Burbank, 1962 ▸; Christe *et al.*, 1993 ▸). A similar coordination environment has not been observed for sodium yet, even for the related sodium hy­dro­gen fluorides (Ivlev *et al.*, 2017 ▸). The sodium hy­dro­gen fluorides also consists of μ-HF-linked polyhedra, such as the potassium hydrogen fluorides (Coyle *et al.*, 1969 ▸, 1970 ▸).
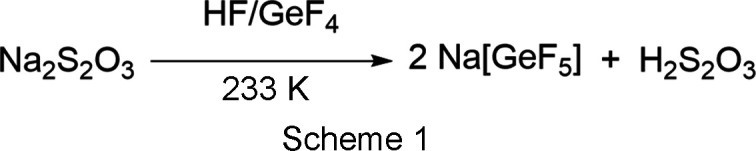


There is a rich structural diversity of [GeF_6_]^2−^-based anions which can be classified in analogy to silicates. The main differences are the octa­hedral coordination of germanium and connections of [GeF_6_]^2−^ units *via* corners and edges. The most common anions are isolated, such as [GeF_6_]^2−^ (neso), [Ge_2_F_10_]^2−^ (soro) or [Ge_3_F_16_]^4−^. Octa­hedra chains of the anion can also be linked *via cis* or *trans* linkage, *i.e.* {[GeF_5_]^−^}_*n*_, in analogy to inosilicates or can even form loop-branched chains, *i.e.* {[Ge_4_F_19_]^3−^}_*n*_ (Soltner, 2011 ▸). Na[GeF_5_]·2HF shows the rather rare structure element of *trans*-connected chains of penta­fluoro­germanates, similar to [XeF_5_][GeF_5_], the only representative so far documented by crystal structure analysis (Mallouk *et al.*, 1984 ▸).

## Experimental

**Caution! Note that any contact with the described com­pounds should be avoided. Hydrolysis of GeF_4_ and the synthesized salts forms HF which burns skin and causes irreparable damage. Safety precautions should be taken while handling these com­pounds.** All reactions were carried out by employing standard Schlenk techniques on a stainless steel vacuum line. The syntheses of the salts were performed using FEP/PFA (fluoro­ethyl­ene­propyl­ene/perfluoralk­oxy) reactors with stain­less steel valves.

### Synthesis and crystallization

Anhydrous hy­dro­gen fluoride (80.04 mg, 4.0 mmol) and germanium tetra­fluoride (297.16 mg, 2.0 mmol) were con­den­sed into an FEP reactor. The solution was warmed to 233 K and thoroughly mixed for 5 min. Sodium thio­sulfate (158.11 mg, 1.0 mmol) was added to the superacid after freezing it at liquid nitro­gen temperature, and the solution was warmed to 233 K again and thoroughly mixed for 5 min. The volatile com­ponents were removed over a period of 12 h *in vacuo* at 195 K. The product was obtained as colourless crys­tals in qu­anti­tative yield.

### Crystal structure refinement

**Figure 1:**
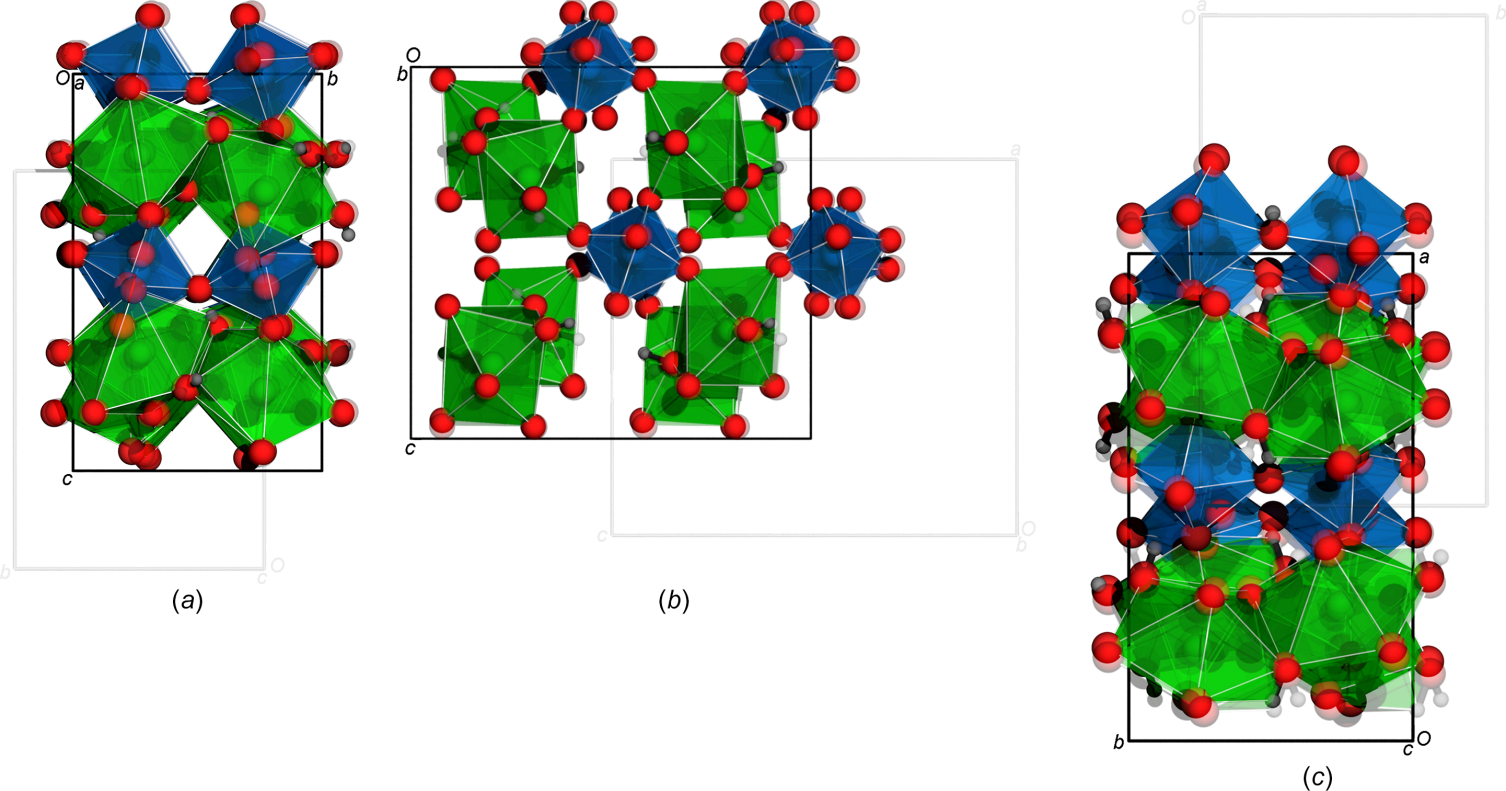
Overlay of the structural models in *Pca*2_1_ and *Pbca* (light-grey unit cell, lighter atoms), viewed along (*a*) the *a* axis, (*b*) the *b* axis and (*c*) the *c* axis. Only H and F atoms show distinct differences in their respective positions, justifying the noncentrosymmetric model.

Basic crystallographic data and details of the data collection and structure refinement are summarized in Table 1 ▸. The positions of the H atoms in the structure were localized in the difference Fourier map and refined without any restrictions (Table 1 ▸). Symmetry checks by *ADDSYM* (Spek, 2001 ▸, 2003 ▸; Le Page, 1988 ▸) supported the space groups *Pbca* and *Pca*2_1_ when regarding the heavy-atom arrangement; however, the noncentrosymmetric space group was only supported when taking the F and H atoms into account, as shown in Fig. 1 ▸. In contrast to the H and F atoms, the Na and Ge atoms contribute to hypersymmetry. The structure was refined as an inversion twin.

The calculated moiety formula was adjusted from ‘F20 Ge4, 8(F H), 4(Na)’ with *Z* = 2 to ‘Na Ge F5, 2(F H)’ with *Z* = 8, since the space group is ortho­rhom­bic and all atoms occupy the general position 4*a*. Due to symmetry, it can also be seen that the chains of octahedra are not isolated [Ge_2_F_10_]^2−^ but instead {[GeF_5_]^−^}_*n*_ units.

### Analysis

**Figure 2:**
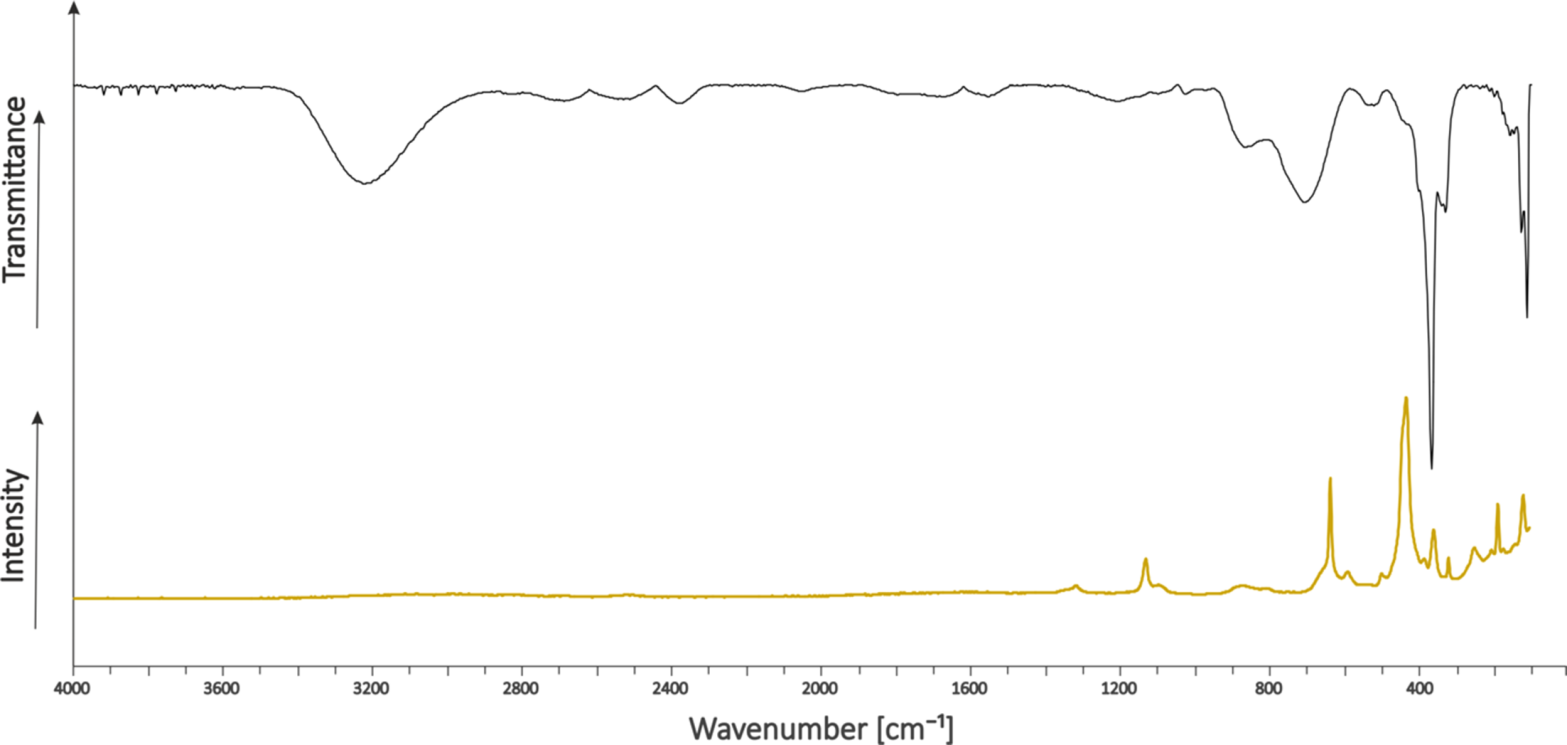
Vibrational spectra of Na[GeF_5_]·2HF, showing IR (top) and Raman (bottom).

The product was further analysed by low-temperature vibrational spectroscopy in order to confirm the conformation of the fluoro­germanate anion. IR spectroscopic investigations were carried out with a Bruker Vertex-80V FT–IR spec­trom­eter using a cooled cell with a single-crystal CsBr plate on which small amounts of the sample were placed (Bayersdorfer *et al.*, 1972 ▸). For the Raman measurements, a Bruker MultiRam FT–Raman spec­trom­eter with Nd:YAG laser excitation (λ = 1064 nm) was used. The measurement was performed after transferring the sample into a cooled (77 K) glass cell under a nitro­gen atmosphere and subsequent evac­u­a­tion of the glass cell. The low-temperature spectra are depicted in Fig. 2 ▸.

**Figure 3:**
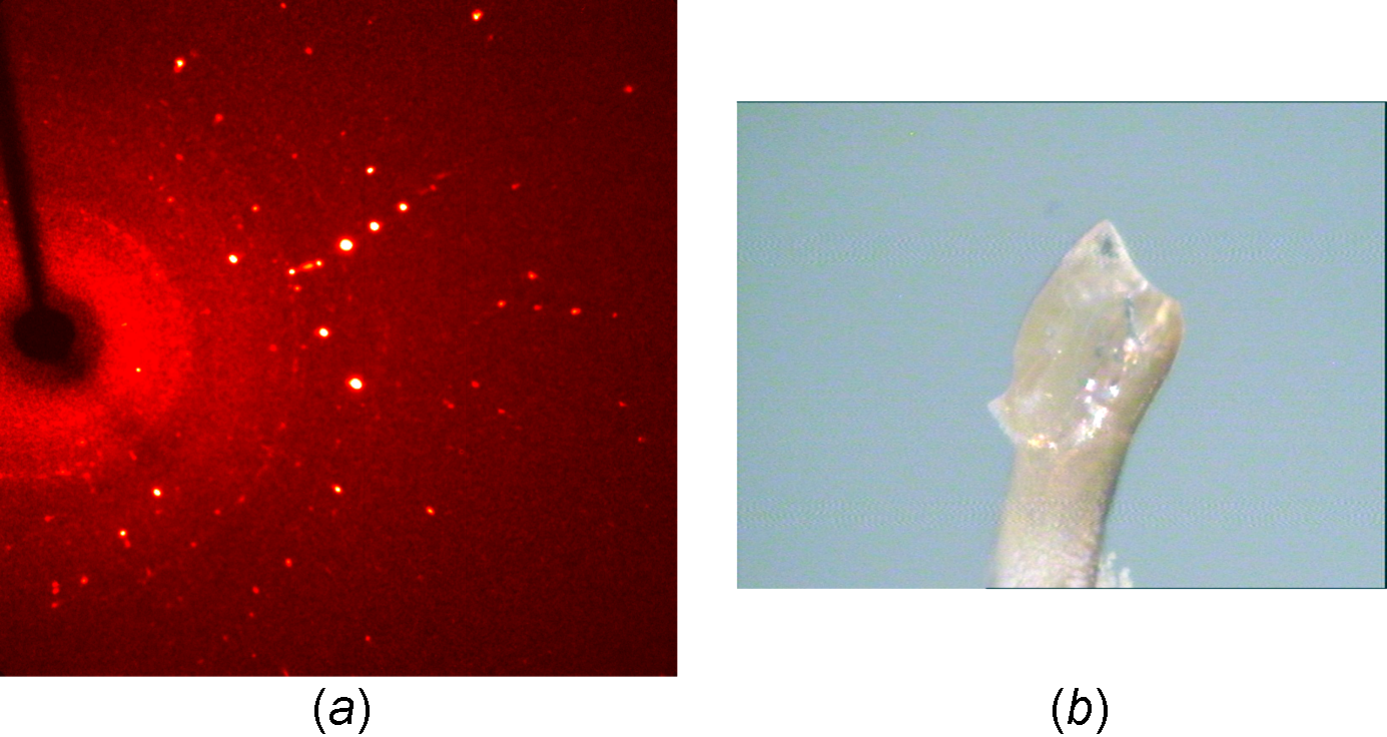
(*a*) Diffraction pattern and (*b*) the prepared single crystal on a polyamide loop of the micromount.

Single crystals of Na[GeF_5_]·2HF suitable for single-crystal diffraction analysis were selected under a stereomicroscope in a cooled nitro­gen stream. The single crystal was prepared on a stainless steel polyamide micromount (see Fig. 3 ▸) and data collection was performed at 117 K on a Xcalibur dif­frac­tom­eter system (Rigaku Oxford Diffraction). For details of the data collection and treatment, as well as of the structure solution and refinement, see the supporting information.

Decom­position of the product was already identified at 238 K by detecting the development of vapour pressure with temperature.

## Results and discussion

### Vibrational spectroscopy

The Raman spectra show a broad line (712–600 cm^−1^) appearing at 665 cm^−1^ for the terminal Ge—F vibration of the [GeF_5_]^−^ anion (654 and 622 cm^−1^) [the frequencies in parentheses are from Mallouk *et al.* (1984 ▸)]. The Ge—F stre­tching vibrations of the 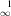
[GeF_5_]^−^ chain appear at be­tween 536 and 524 cm^−1^ (526 and 518 cm^−1^). The bands at 388 (381), 336 (339) and 329 cm^−1^ (331 cm^−1^) can be assigned to the square-plane angle deformation modes. These vibrations are similar to the values reported by Mallouk *et al.* (1984 ▸), but the data suffers from overlap in the fingerprint area.

The IR spectra reveal the existence of hy­dro­gen fluoride by its rotation bands at high wavenumbers (3921, 3879, 3834 3788, 3742, 3693 and 3643 cm^−1^). In addition, the [NaF_7_] polyhedra show bands similar to the structurally related IF_7_ (Christe *et al.*, 1993 ▸) that can be found at 758 (746), 657 (670), 403 (425), 374 and 358 cm^−1^ (365 cm^−1^).

The lines at 1342 [ν(SO_3_)] and 1158 cm^−1^ [ν(SO_2_)] are due to the decom­position of the solvent (H_2_S_2_O_3_) according to Scheme 2, as are the bands at 3269 [ν(OH)], 1067 [ν(SO)] and 916 cm^−1^ [ν(SF)]. It can be assumed that the sulfur dioxide released by the decom­position of thio­sulfuric acid reacts with excess hy­dro­gen fluoride to form fluoro­sulfinic acid, as well as traces of polythio­nic acids, as reported in the literature (Hopfinger *et al.*, 2018 ▸).
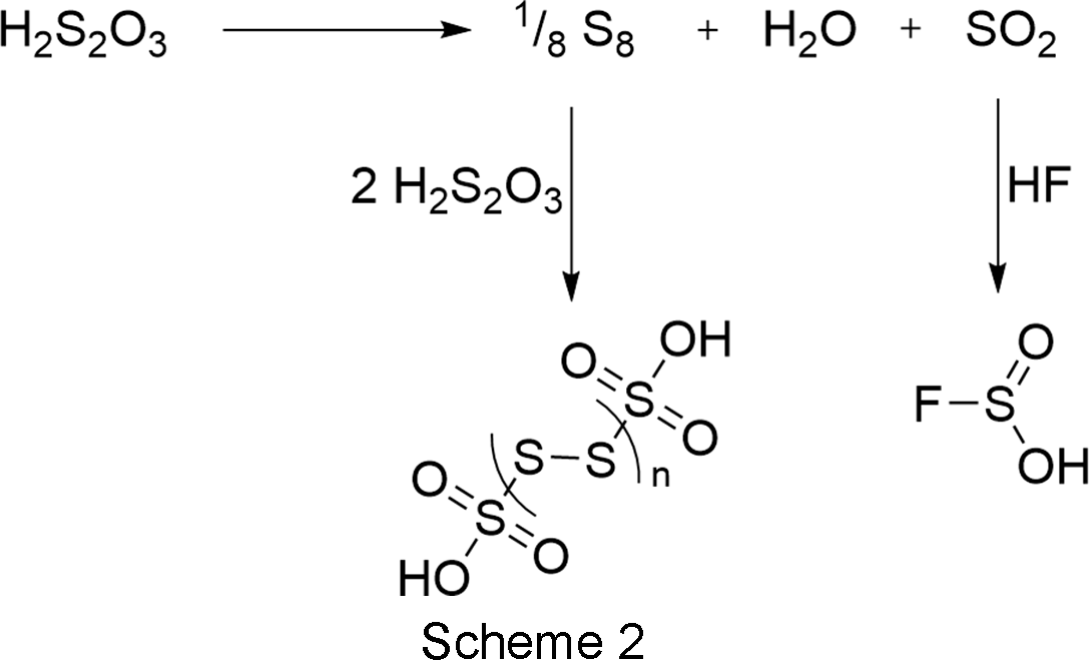


Since the structural chemistry of fluoro­germanates has not been fully understood, other anions, as calculated by Soltner (2011 ▸), were com­pared with the observed data. Therefore, vibrations were also assigned to [GeF_5_]^−^ in accordance with the literature. The final assignments of vibrations for Na[GeF_5_]·2HF are listed in Table 2 ▸.

### Crystal structure

**Figure 4:**
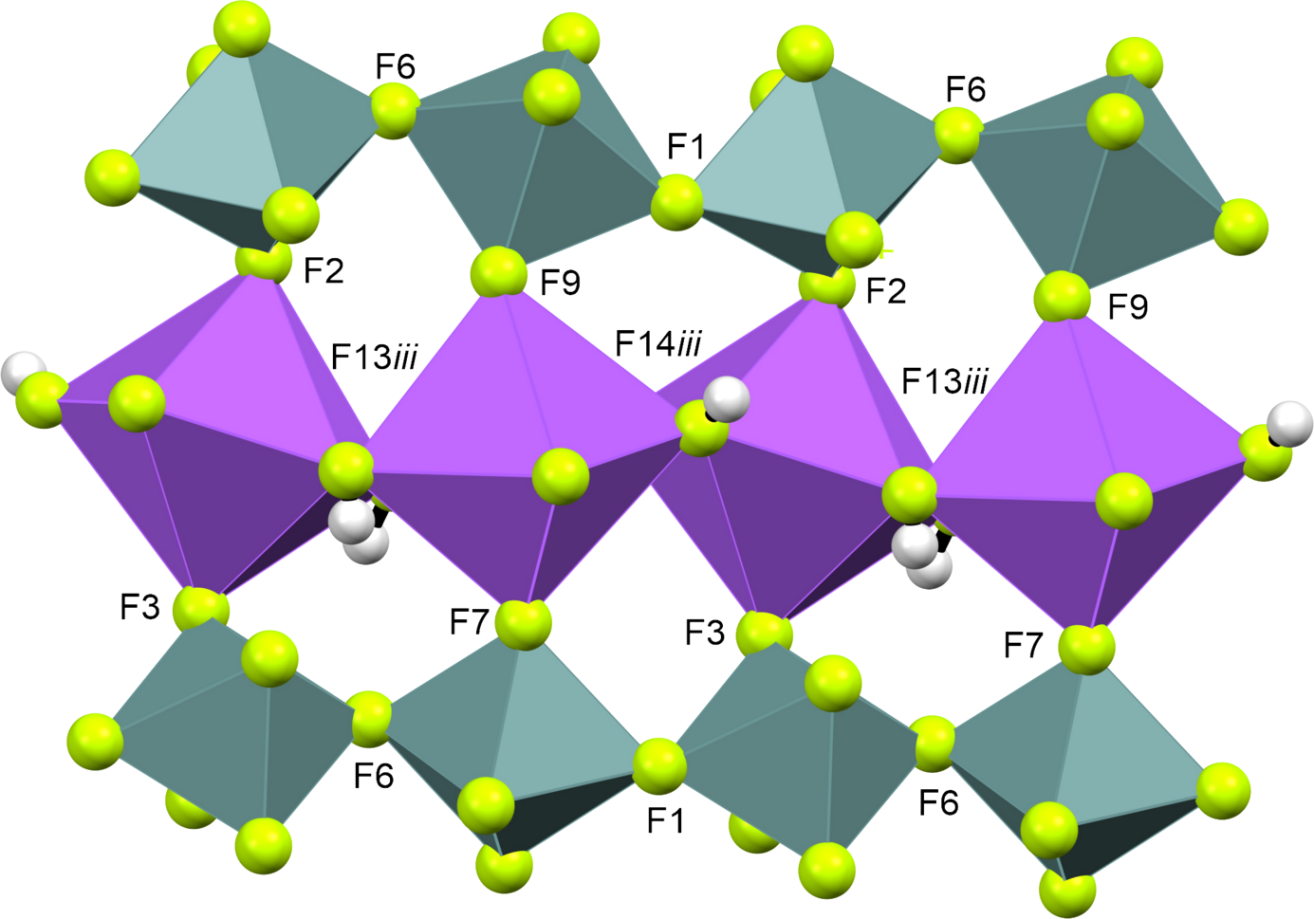
Structural cut-out of the crystal structure of Na[GeF_5_]·2HF, viewed along the *a* axis. Na-centred and Ge-centred polyhedra are shown in purple and grey, respectively. [Symmetry codes: (i) −*x*, −*y*, *z* − 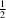; (ii) *x* + 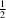, −*y*, −*z* + 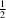; (iii) −*x* + 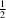, *y*, *z* + 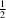.]

**Figure 6:**
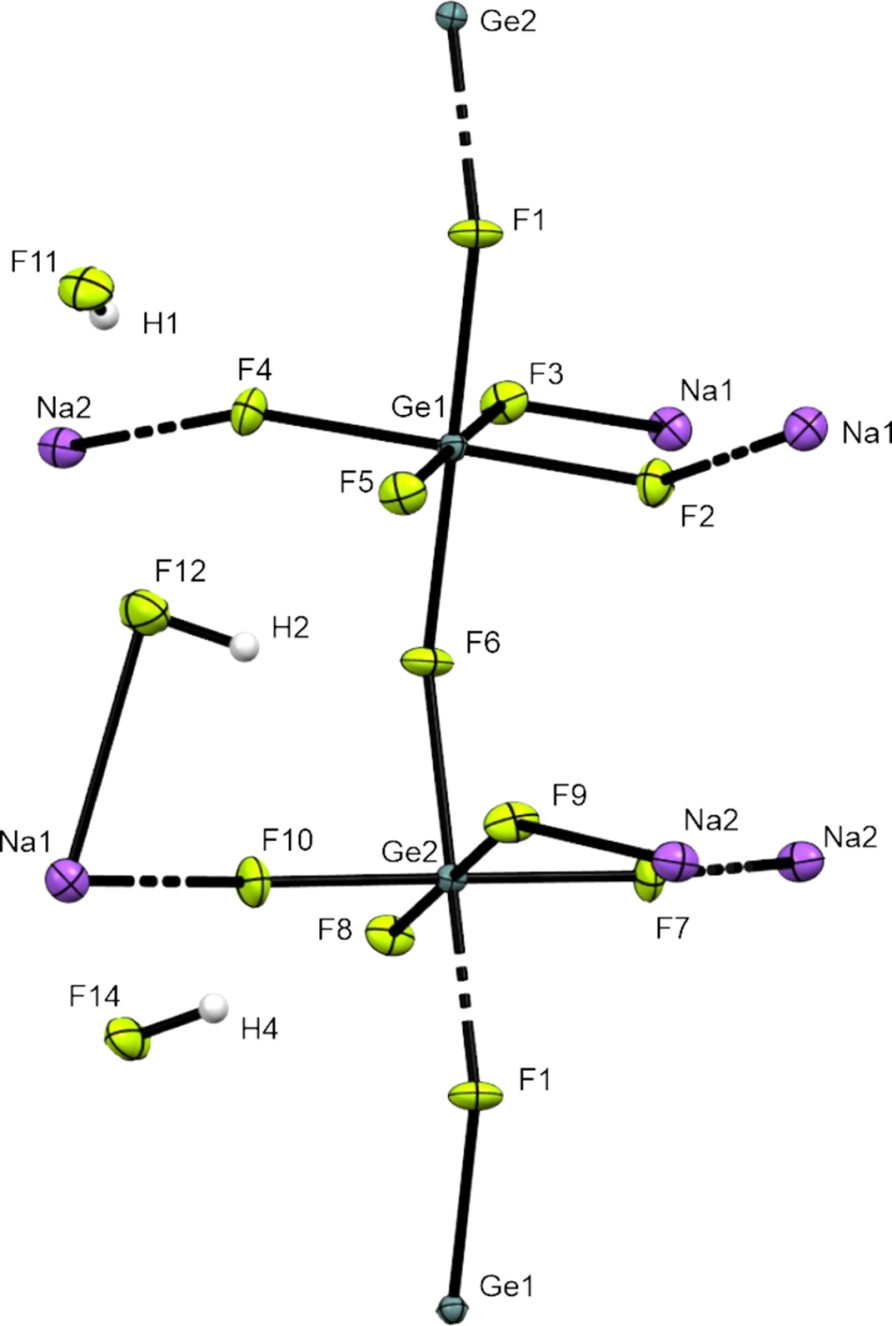
The coordination environments of Ge1 and Ge2. Displacement ellipsoids are displayed at the 50% probability level.

In the 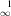
[GeF_5_]^−^ chains (Figs. 4 ▸ and 5 ▸), the *trans*-connected [GeF_6_]^2−^ octa­hedra are tilted 28.94° with respect to each other, and the octa­hedra are connected by atoms F1 and F6 (Fig. 6 ▸). The chains are arranged along the *b* axis and bent at Ge—F—Ge by 146.19°, forming zigzag chains. The octa­hedra are formed by atoms F1–F6 for Ge1 and F6–F10 for Ge2. The non­bridging Ge—F bonds are in the range 1.736 (2)–1.7719 (17) Å, in contrast to the bridging F atoms, which have a range of 1.8711 (15)–1.9020 (16) Å between Ge1 and Ge2. The atomic coordinates, anisotropic displacement parameters and inter­atomic distances and angles are com­piled in the supporting information. The [GeF_5_]^−^ units in Na[GeF_5_] show similar Ge—F bond lengths to those in [XeF_5_][GeF_5_], but are slightly different due to distortion (Table 3 ▸).

**Figure 7:**
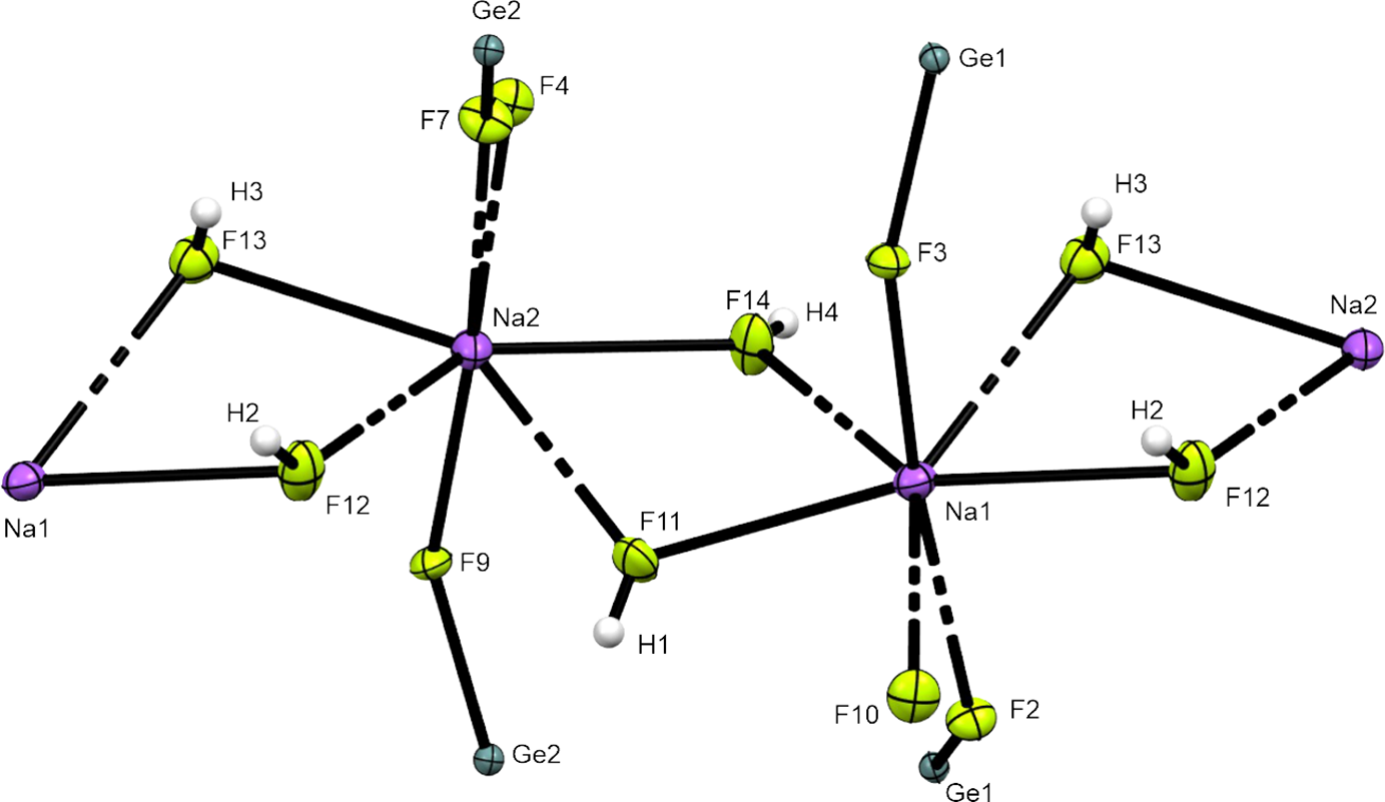
The coordination environments of Na1 and Na2. Displacement ellipsoids are displayed at the 50% probability level.

The sodium ions exhibit an unusual distorted penta­gonal bipyramidal coordination. The coordination spheres of Na1 and Na2 are built up from atoms F2–F4 belonging to one *trans*-penta­fluoro­germanate anion and from F7, F9 and F10 from the second *trans*-penta­fluoro­germanate anion, and four F atoms (F11–F14) belonging to HF mol­ecules (Fig. 7 ▸). The μ-F-bridged Na1- and Na2-centred polyhedra are *trans*-edge-linked, forming an infinite tilted chain extended along the *b* axis. The distances between Na1 and Na2 are 3.906 (2) and 3.934 (2) Å, respectively, and the Na—F distances range from 2.271 (2) to 2.610 (3) Å. Therefore, Na[GeF_5_]·2HF displays similar Na—F distances, but with higher deviations, com­pared to NaH_4_F_5_ (Table 4 ▸). The different distances of the μ-HF bridges leads to distortion of the penta­tagonal bipyramid by the germanium chains.

Two very strong hy­dro­gen bonds are formed, namely, F12—H2⋯F5 [2.499 (3) Å] and F14—H4⋯F8 [2.483 (3) Å]. Two medium-strong hy­dro­gen bonds form the connections F11—H1⋯F4 [2.661 (3) Å] and F13—H3⋯F2 [2.637 (3) Å]. Weaker inter­actions are F13—H3⋯F7 [3.007 (3) Å], F11—H1⋯F7 [2.895 (3) Å], F11—H1⋯F10 [2.870 (3) Å], F14—H4⋯F5 [3.105 (3) Å] and F12—H2⋯F8 [3.173 (3) Å]. The given distances are derived from F⋯F interatomic distances. In accordance with the criteria given by Jeffrey (1997 ▸), the assignment of weak/strong hy­dro­gen bonds shows short and directed contacts for strong and longer and nondirectional contacts for weaker hy­dro­gen bonds.

## Conclusion

Thio­sulfuric acid could not be protonated in the superacidic system HF/GeF_4_ as intended. However, thio­sulfuric acid proved to be a solvent for the crystallization of new *A*[Ge_*x*_F_*y*_]_*z*_ salts (*A* = alkali or alkaline-earth metals) due to the balanced acidity, volatility and extraordinary solubility of fluorine-con­taining metal salts. By exploiting this method, new structures of alkali or alkaline-earth fluoro­germanates might be­come accessible.

Expanding the gaps between the infinitive chains might result in new structures or might cause conformational changes in the fluoro­germanate chains. Following this procedure, the structural chemistry of fluoro­germantes could be­come more com­prehensive. In analogy to silicates, ring form­ation might be observed in com­pounds with large low-charged cations.

It may also be possible to synthesize Na[GeF_5_] in a sim­plified reaction of sodium fluoride in HF/GeF_4_ and it may be possible to improve the spectroscopic data, as decom­position of the solvent (H_2_S_2_O_3_) could be avoided. Since the investigations were originally aimed at the protonation of thio­sulfuric acid, no futher attempt was made to figure out whether the presence of thio­sulfuric acid is necessary as a solvent or if the reaction could also just succeed in anhydrous hy­dro­gen fluoride. As the solubility of sodium hy­dro­gen fluorides increases drastically in anhydrous hy­dro­gen fluoride with higher hy­dro­gen fluoride content at low temperature, it can be expected that without additional solvent the reaction needs to be heated to homogenize the product. Otherwise a mixture of NaH_4_F_5_ and NaF may be obtained reacting with the Lewis acid GeF_4_, leading to a mixture of different Na[GeF_5_]·*n*HF.

Furthermore, the levelling effect of sodium salts could be shown for GeF_4_-based systems, in analogy to BF_3_ decreasing Lewis acidity under the formation of sodium salts.

## Supplementary Material

Crystal structure: contains datablock(s) global, I. DOI: 10.1107/S2053229624006338/yd3042sup1.cif

Structure factors: contains datablock(s) I. DOI: 10.1107/S2053229624006338/yd3042Isup2.hkl

CCDC reference: 2366255

## Figures and Tables

**Figure 5:**
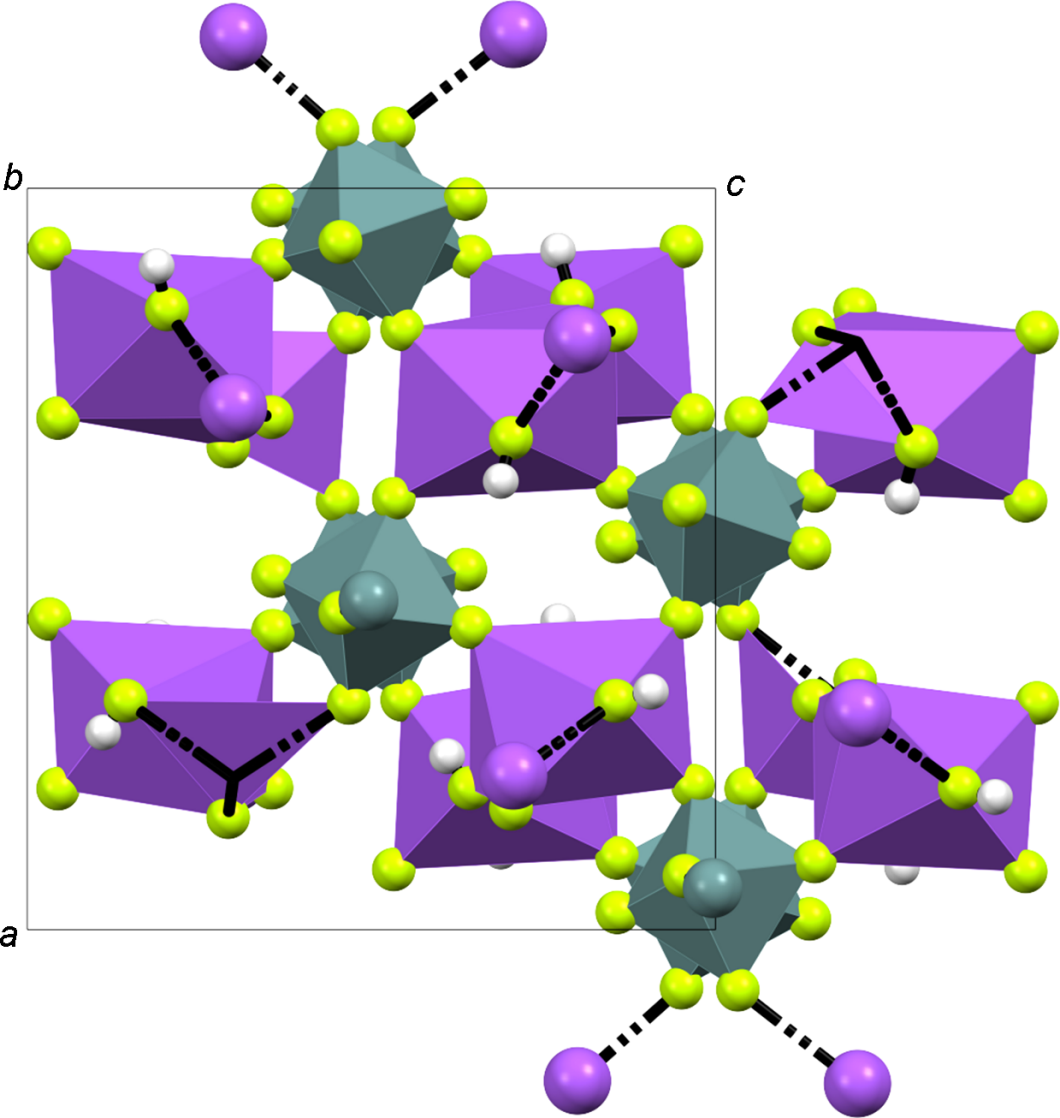
The crystal structure of Na[GeF_5_]·2HF, viewed along the *b* axis. Na-centred and Ge-centred polyhedra are shown in purple and grey, respectively.

**Table 1:** Experimental details

Crystal data	
Chemical formula	Na[GeF_5_]·2HF
*M*_r_	230.60
Crystal system, space group	Orthorhombic, *Pca*2_1_
Temperature (K)	117
*a*, *b*, *c* (Å)	12.3786 (3), 7.2189 (2), 11.4969 (3)
*V* (Å^3^)	1027.36 (5)
*Z*	8
Radiation type	Mo *K*α
μ (mm^−1^)	6.12
Crystal size (mm)	0.39 × 0.27 × 0.20

Data collection	
Diffractometer	Rigaku Xcalibur Sapphire3
Absorption correction	Multi-scan (*CrysAlis PRO*; Rigaku OD, 2020 ▸)
*T*_min_, *T*_max_	0.566, 1.000
No. of measured, independent and observed [*I* > 2σ(*I*)] reflections	19491, 3147, 2948
*R*_int_	0.020
(sin θ/λ)_max_ (Å^−1^)	0.714

Refinement	
*R*[*F*^2^ > 2σ(*F*^2^)], *wR*(*F*^2^), *S*	0.016, 0.041, 1.06
No. of reflections	3147
No. of parameters	180
No. of restraints	1
H-atom treatment	All H-atom parameters refined
Δρ_max_, Δρ_min_ (e Å^−3^)	0.35, −0.42
Absolute structure	Refined as an inversion twin
Absolute structure parameter	0.482 (13)

Computer programs: *CrysAlis PRO* (Rigaku OD, 2020 ▸), *SHELXT* (Sheldrick, 2015*a* ▸), *SHELXL2018* (Sheldrick, 2015*b* ▸), *ORTEP-3 for Windows* (Farrugia, 2012 ▸) and *PLATON* (Spek, 2020 ▸).

**Table 2:** Vibrational assignments for Na[GeF_5_]·2HF (frequencies in cm^−1^) Abbreviations for IR intensities: *v* = very, *s* = strong, *m* = medium, *w* = weak. Experimental Raman activities are relative to a scale of 1 to 100.

Raman	IR	Raman (literature)	IR (literature)	Assignment
	758 (*w*)		746 (*s*)	ν_as_ NaF_2_ axial
		676 (2)		ν_s_ NaF_2_ axial
665 [100, *vs* (broad)]		654		ν_s_ [GeF_5_]_*n*_ terminal
		635 (10)		ν_s_ NaF_5_ axial
		622		ν_as_ [GeF_5_]_*n*_ terminal
	657 (*w*)		670 (*vs*)	ν_as_ NaF_5_ equatorial
	596 (*s*)	596 (0.2)		mixture δ sciss of NaF_5_ in-plane
536 (18, *w*)		526		ν [GeF_5_]_*n*_ chain
524 (19, *w*)		518		ν [GeF_5_]_*n*_
	403 (*w*)		425 (*vs*)	δ_as_ NaF_5_ in-plane
388 (24, *w*)		381		δ [GeF_5_]_*n*_ equatorial
	374 (*w*)		365 (*s*)	δ umbrella NaF_5_ equatorial
	358 (*m*)		365 (*s*)	δ umbrella NaF_5_ equatorial
336 (26, *w*)		339		δ [GeF_5_]_*n*_
329 (26, *w*)		329		δ [GeF_5_]_*n*_

**Table 3:** Structural com­parison of Ge—F bond lengths (Å)

Na[GeF_5_]·2HF		[XeF_5_][GeF_5_]		[(Me_2_OH)_2_][Ge_2_F_10_]	
(This work)		(Mallouk *et al.*, 1984 ▸)		(Soltner, 2011 ▸)	
Ge1—F1	1.8752 (15)	Ge—F1	1.745 (2)	Ge1—F1	1.7918 (12)
Ge1—F2	1.7719 (17)	Ge—F2	1.745 (2)	Ge1—F2	1.7393 (12)
Ge1—F3	1.741 (2)	Ge—F3	1.890 (1)	Ge1—F3	1.7450 (12)
Ge1—F4	1.770 (2)			Ge1—F4	1.7426 (12)
Ge1—F5	1.750 (2)			Ge1—F5	1.9128 (12)
Ge1—F6	1.8711 (15)			Ge1—F5′	1.9515 (12)
Ge2—F7	1.751 (2)				
Ge2—F8	1.765 (2)				
Ge2—F9	1.736 (2)				
Ge2—F10	1.745 (2)				
Ge2—F1^i^	1.8923 (15)				

Symmetry code: (i) *x*, *y* + 1, *z*.

**Table 4:** Structural com­parison of Na—F inter­atomic distances (Å)

Na[GeF_5_]·2HF		NaH_4_F_5_ (Ivlev *et al.*, 2017 ▸)	
Na1—F3	2.271 (2)	Na—F2	2.4337 (5)
Na1—F10^ii^	2.333 (3)	Na—F2	2.5104 (4)
Na1—F12	2.337 (2)		
Na1—F2^iii^	2.348 (3)		
Na1—F11	2.359 (2)		
Na1—F14^iv^	2.385 (2)		
Na1—F13^iii^	2.610 (3)		
Na2—F9	2.236 (2)		
Na2—F7^v^	2.252 (3)		
Na2—F14	2.334 (2)		
Na2—F13	2.373 (2)		
Na2—F12^v^	2.431 (2)		
Na2—F11^vi^	2.500 (3)		
Na2—F4^vii^	2.557 (3)		

Symmetry codes: (ii) −*x* + 1, −*y* + 1, *z* + 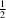; (iii) −*x* + 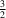, *y*, *z* + 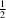; (iv) −*x* + 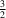, *y* − 1, *z* + 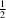; (v) −*x* + 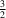, *y*, *z* − 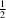; (vi) −*x* + 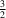, *y* + 1, *z* − 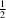; (vii) −*x* + 1, −*y* + 1, *z* − 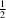.
